# Designing and validating the Dubai Tool for Developmental Screening (DTDS)

**DOI:** 10.3389/fped.2022.924017

**Published:** 2022-08-22

**Authors:** Fatma Mohammad Alolama, Haitham Mahmoud Mohammad, Idris Helal Alhmid, Hanan Mohammed Alhammadi

**Affiliations:** ^1^Child Health Section, Specialized Programs Services Department, Primary Health Care Sector, Dubai Health Authority, Dubai, United Arab Emirates; ^2^Medical Affairs Department, Primary Health Care Sector, Dubai Health Authority, Dubai, United Arab Emirates

**Keywords:** child development, developmental screening, screening tool, developmental delay, motor development, speech development, social emotional development, problem solving self-help development

## Abstract

**Background:**

Early detection of developmental problems is vital for facilitating early access to targeted intervention and augmenting its beneficial outcomes. Standardized developmental screening tools are known to enhance detection rates of developmental problems compared to clinical judgment alone and are widely recommended to be used in infants and young children. Most of the available developmental screening tools have been developed in Western countries. Many of their items may not be suitable for other cultures while others are expensive. Currently, none of the developmental screening tools have been validated in the United Arab Emirates (UAE), with only a few available in the Arabic language.

**Objective:**

To create and validate a developmental screening tool, in both English and Arabic, that is simple, quick to use, and culturally relevant to the United Arab Emirates (UAE) child population aged 9–48 months.

**Methods:**

The available literature was used to create a list of developmental milestones in five domains for children aged 9–48 months, divided into seven age groups. The selected milestones were used to create questionnaires in both English and Arabic, which were pilot tested twice. Each time, the results were analyzed and used to select, modify, and rephrase questions. Validation of the Dubai Tool for Developmental Screening (DTDS) was done against Parents' Evaluation of Developmental Status (PEDS) as a gold standard instrument. The DTDS and PEDS were administered cross-sectionally to parents of 1,400 children in seven age groups. Sensitivity, specificity, and kappa agreement of the DTDS compared with PEDS were calculated.

**Results:**

The DTDS had a sensitivity of 100% in four age groups and 75–78% in the other three age groups. Specificity ranged from 96 to 99% across all age groups. The kappa measure showed substantial agreement in five age groups, a moderate agreement in one age group, and a fair agreement in one age group.

**Conclusions:**

The DTDS is a valid screening tool for early identification of developmental delays and disabilities in early childhood.

## Introduction

Pediatricians and other primary care physicians frequently see children with abnormal development and behavior. Disordered development and behavior affect about 12–16% of American children below 5 years of age (1, 2). Early identification of developmental disorders should lead to a thorough evaluation, and hence proper early intervention (3). However, data from the United States revealed that only approximately one out of three children with developmental abnormalities are recognized prior to school age, while others do not benefit from early intervention services (4–6). One of the main factors contributing to low detection rates is reliance on clinical judgment alone (7), which can miss up to 45% of eligible children for early intervention services (8). The American Academy of Pediatrics recommends regular screening for developmental disorders using a standardized tool at 9, 18, and 30 (or 24) months of age and at every well-child visit whenever concerns regarding the development are raised during surveillance (2). Developmental screening tools can accurately detect developmental delays (9). However, screening for developmental delays in normal clinical practice is fraught with difficulties such as time limitations, shortage of required subspecialties for referral, limited training and confidence of physicians in managing children with developmental abnormalities, and difficulties in finding the perfect screening tool (10–14). Most of the available developmental screening tools have been developed in Western countries. Many of their items may not be suitable for other cultures (15, 16), while others are expensive. The tools currently used in the Middle East and North Africa (MENA) region are mainly translated from the globally used tools such as Ages and Stages Questionnaire (ASQ), Denver Developmental Screening Test and Parents' Evaluation of Developmental Status (PEDS) (17). PEDS is one of the most extensively evaluated parent completed tools. F. P. Glascoe created this tool in Tennessee, USA in 1997 (18). It is a 10-item questionnaire that captures parents' concerns about their child's developmental skills, including fine and gross motor, expressive and receptive language, behavior, social, self-care, and learning (19). It is applicable for children aged 0–8 years and takes 2–5 min to complete. PEDS has been validated in many studies. According to recent validation studies from the United States, PEDS has a sensitivity of 91–97% and a specificity of 73–86% (20). It is available in Arabic and English, among many other languages. Ages and Stages questionnaire (ASQ) is another widely used screening tool created in high income country. It was designed and developed by Squires, Bricker and Twombly (21) at the University of Oregon, USA. The Ages and Stages questionnaire third edition (ASQ3) is a parent completed tool, consisting of 21 questionnaires (30-items each) targeting the age of 2–60 months can be completed by parents in 12–18 min. The overall sensitivity and specificity of ASQ are 75% and of 86% respectively. The ASQ has been translated into several languages including Arabic language (22). In a systemic review by Fischer VJ et al. (23), fourteen developmental screening tools created in low- and middle-income countries (LMIC) were included. Most of these tools were locally developed, but not validated across different cultures. Only 4 of them expressed sensitivity and specificity >80%. These tools are: Disability Screening Schedule (DSS) (24), Ten Questions Screen (TQS) (25) for Childhood Disability, Guide for Monitoring Child Development (GMCD) (15), and Malawian Developmental Assessment Tool (MDAT) (26). The Egyptian developmental screening chart (EDSC); to our knowledge; is the first tool to be developed in Arab countries. It was developed by El Shafie AM et al. in 2020. The EDSC can be applied to children 0–30 months old. Initially a Z-score chart for motor and mental development was created, then the EDSC validity was tested against ASQ3 with an overall sensitivity of 84.38% and specificity of 98.36% (27). When looking to the methods followed to create different available screening tools, 2 models were identified. The first is to study the normal infants and children to create the norms of development in the community. Bayley Scales of Infant and Toddler Development (28), Baroda development screening test (29), Denver Developmental Screening Test II (30), and Trivandrum Development Screening Chart (31) are examples of this model. In the second model tools are prepared through review of literature including other tools, then pilot test for cultural appropriateness and modify accordingly. Examples of this model are: The Disability Screening Schedule (DSS) (24), Developmental Assessment Tool for Anganwadis (32)**, **The Ten Questions Screen (25), The Kilifi Developmental Inventory (33), and The Malawian Developmental Assessment Tool (26).

Currently, none of the developmental screening tools have been validated in the United Arab Emirates (UAE), and few are available in the Arabic language.

Therefore, the aim of this study was to create and validate a parent-completed developmental screening tool for infants and children aged 9–48 months in both the English and Arabic languages. We aimed for it to be simple, quick to administer and score, and culturally relevant to the UAE population.

## Methods

This study was conducted from December 2018 to January 2020 at child health clinics located in twelve primary health care centers of the Dubai Health Authority (DHA) distributed throughout the Emirate of Dubai. The Dubai Scientific Research Ethics Committee (DSREC), DHA, authorized the study protocol. The study was divided into two stages: the first was to design the tool, and the second was to validate it.

### First stage: Design of the Dubai Tool for Developmental Screening

The first stage of the study consisted of five steps.

#### Step 1: Data collection and defining milestones

A working group was created, consisting of child health section pediatricians in the DHA. The working group reviewed the available literature to generate a list of developmental milestones for infants and children aged 9–48 months, including five developmental domains: speech and language, social and emotional, problem solving and self-help, gross motor, and fine motor. The age period was divided into seven age stages: 9–11 months, 12–14 months, 15–17 months, 18–23 months, 24–29 months, 30–35 months, and 36–48 months. For each age group, five developmental milestones in each domain were selected, taking into consideration community appropriateness and applicability.

#### Step 2: Creating questionnaires

The chosen milestones were rewritten in the simplest English language possible, with a reading level of no more than fifth grade. Each milestone was transformed into a “Yes” or “No” question beginning with “Does your child.” The questions were translated and formulated in Arabic language using the forward-backward translation method. First the questions were translated by one of the Arabic speaking authors of the tool and reviewed by 2 others. The revised Arabic version of questionnaires was independently translated backward into English by a professional translator who was blinded to the original questionnaires. The tool authors matched the backward translation with the original English questionnaires and revised the Arabic translation accordingly. The Arabic version of questionnaires was finalized ensuring conceptual equivalence to the original English version.

#### Step 3: Pilot study

The first draft of questionnaires was then piloted on parents of 420 infants and children. The parents of 60 children in each age group were recruited from the primary health care center child health clinics of the DHA; of them, 30 were given the English version and 30 were given the Arabic version. The inclusion criteria were that the available parent parents must be living with the child, have at least a primary education, and speak English fluently (for the English version) or speak Arabic fluently (for the Arabic version). Children with a history of prematurity, and children diagnosed with developmental delay or disability were excluded. After obtaining written consent, each parent was asked to rate each question on a scale of 1–3 for its understandability and applicability (for understandability: 1 was coded as hard to understand, 2 was coded as somewhat hard to understand, and 3 was coded as easy to understand; for applicability: 1 was coded as not applicable to my child; 2 was coded as somewhat applicable to my child, and 3 was coded as highly applicable to my child). The results were collected and analyzed. Out of five questions in each domain, three questions with the best score were selected to form the second draft.

#### Step 4: Re-piloting

Using the second draft, the pilot study was repeated on parents of 420 infants and children. The same inclusion/ exclusion criteria and distribution of participants used for Step 3 were applied. The results were collected and analyzed. Questions that were hard to understand or non-applicable were changed or replaced. Few questions needed to be modified to create the third draft.

#### Step 5: Design of the DTDS

At this stage, there were seven questionnaires for seven age groups of children between 9 and 48 months old.

- Group A: age range from 9 months to 11 months and 30 days
- Group B: age range from 12 months to 14 months and 30 days
- Group C: age range from 15 months to 17 months and 30 days
- Group D: age range from 18 months to 23 months and 30 days
- Group E: age range from 24 months to 29 months and 30 days
- Group F: age range from 30 months to 35 months and 30 days
- Group G: age range from 36 months to 48 months

Each questionnaire consists of 15 domain-specific questions (three in each domain). In all questions, the response is either “Yes” or “No.” with “Yes” as the typical response to all questions. A response of yes was scored as 1 while a response of no was scored as 0. Domain scoring is adopted, with a passing score of 3 or 2 in each domain and a fail score of 0 or 1. Achieving a passing score in all domains means negative screening, while getting a fail score in one or more domains means positive screening. Adjusted age is used for premature children up to the age of 24 months to select the appropriate questionnaire.

### Second stage: Validation of the DTDS

The DTDS validity was tested against gold standard instruments, which are the Parents' Evaluation of Developmental Status (PEDS) tools, including the Parents' Evaluation of Developmental Status (PEDS) and the Parents' Evaluation of Developmental Status: Developmental Milestones (PEDS:DM). A cross-sectional study was conducted to examine the developmental screening results—negative screening (pass) or positive screening (fail)—of the DTDS compared with the PEDS tools for the same sample of participants.

#### Measures

##### PEDS tools

PEDS questionnaire consists of ten open-ended questions that captures parents' concerns about their child's developmental skills. The scoring relies on classifying parents' concerns as per their child's age into predictive and non-predictive concerns. Based on that, PEDS provides an interpretation guide of five paths: path A, when parents have two or more predictive concerns; path B, when parents have one predictive concern; path C, when parents have non-predictive concerns; path D, when there are parental difficulties communicating; and path E, when parents have no concerns.

PEDS can also be applied in combination with PEDS:DM, which is a set of parent-completed questionnaires designed to determine whether specific developmental milestones have been met. Each questionnaire consists of six to eight questions for each age stage. The questions include six developmental domains: fine motor, gross motor, receptive language, expressive language, behavior and social skills, and self-care and learning (34). It is available for children from birth to 8 years of age. PEDS:DM forms from D to P, which cover the age periods from 9 to 48 months, were used in this study, and adjusted chronological age was used for premature children <24 months of age.

*Pass/fail criteria of the PEDS tools:* PEDS and PEDS:DM scoring and interpretation were done according to the instructions in their respective manuals. PEDS results were scored as positive screening (fail) when the result was path A and as negative screening (pass) when the result was path E. PEDS:DM was applied as a second-stage developmental screen when the result was path B, C, or D. If an infant/a child meets all the milestones in the corresponding PEDS:DM form, the test result is considered negative screening (pass), whereas if one or more milestones were unmet, the result is positive screening (fail). Pass/fail criteria of the PEDS tools are summarized in Table 1.

**Table 1:** Pass and fail criteria of Parents' Evaluation of Developmental Status (PEDS) tools.

**PEDS result**	**Path**	**Pass**	**Fail**
≥2 predictive concerns	A	Non	All
1 predictive concern	B	0 unmet milestones on PEDS:DM	≥1 unmet milestone on PEDS:DM
Non-predictive concern/s	C	0 unmet milestones on PEDS:DM	≥1 unmet milestone on PEDS:DM
Parental difficulties communicating	D	0 unmet milestones on PEDS:DM	≥1 unmet milestone on PEDS:DM
No concerns	E	All	Non

*PEDS:DM, Parents' Evaluation of Developmental Status: Developmental Milestones*.

##### DTDS

The scoring and pass/fail criteria have been described above.

### Participants

We aimed to enroll 200 participants in each age group. The parents/caregivers of infants and children were recruited from those attending child health clinics for routine assessment and immunization. The inclusion criteria were that the available parent or caregiver should be living with the child and have at least a fifth-grade reading ability (Arabic or English). Children with diagnosed developmental delay or disability were excluded. The infants and children who shared in the first stage were not excluded in the second stage of the study.

### Procedure

Thirty-six child health nurses from 12 primary health care centers were trained to administer and score DTDS, PEDS, and PEDS:DM. They were also trained to identify eligible children, to explain the study, and to obtain written consent. The trained child health nurses met parents/caregivers of children during their routine visits to child health clinics. They checked for eligibility, explained the study to them, and obtained their written consent. Both the DTDS and PEDS were handed to parents/caregivers, and they were requested to answer all the questions. The answered questionnaires were scored immediately. Parents/caregivers of children with PEDS path B, C, and D were requested to complete PEDS:DM. The final results of the PEDS tools together with the DTDS were recorded as negative screening (pass) or positive screening (fail).

### Statistical analysis

PASS 16 (PASS 16 Power Analysis and Sample Size Software (2018). NCSS, LLC. Kaysville, Utah, USA, ncss.com/software/pass) was used for sample size calculation in the validation study (35). We calculated the sample size to obtain high sensitivity to reduce the false negative rate. A sample size of 185 produces a two-sided 95% confidence interval with a margin of error 10% when the sample sensitivity is 90% and the prevalence is 20%.We used SPSS Statistics version 24 for Windows (IBM Corp., Armonk, NY, USA) for statistical analysis. Categorical data are presented as count and percent. We measured the validity of the DTDS by calculating the sensitivity and specificity (with 95% confidence intervals) compared with the PEDS tools to identify positive and negative cases (36). The positive predictive value and negative predictive value were calculated as well. We calculated the kappa coefficient (with a 95% confidence interval) to measure the agreement between the DTDS and PEDS results. A kappa value of 0.61–0.8 is considered substantial agreement, and a kappa value of 0.41–0.6 is considered moderate agreement (37). We evaluated construct validity with factor analysis using the SPSS macro to calculate the tetrachoric correlation matrix for binary data (38). We used principle component (PC) factor analysis with VARIMAX rotation. Bartlett's test was significant (p <0.05) for all age groups. We calculated the average pairwise percent agreement between the items within each domain of the DTDS to measure the reliability of each domain in each age group.

## Results

The total number of participants who completed the DTDS, PEDS, and PEDS:DM (when required) was 1,440; the participants were parents of infants and children aged between 9 and 48 months divided into seven age groups (A–G). The demographic characteristics of the participants are summarized in Table 2.

**Table 2:** Demographic information of infants and children who participated in the study.

**Age group**	**Gender**		**Nationality**		**Total**
	**Male (%)**	**Female (%)**	**UAE national (%)**	**Non-UAE national (%)**	
A (9–11 months and 30 days)	112 (55.7%)	89 (44.3%)	61 (30%)	140 (70%)	201
B (12–14 months and 30 days)	107 (53.5%)	93 (46.5%)	51 (25.5%)	149 (74.5%)	200
C (15–17 months and 30 days)	112 (55.7%)	89 (44.3%)	60 (29.9%)	141 (70.1%)	201
D (18–23 months and 30 days)	115 (50.9%)	111 (49.1%)	61 (27%)	165 (73%)	226
E (24–29 months and 30 days)	121 (55.3%)	98 (44.7%)	77 (35.2%)	142 (64.8%)	219
F (30–35 months and 30 days)	93 (48.7%)	98 (51.3%)	64 (33.5%)	127 (66.5%)	191
G (36–48 months)	102 (50.5%)	100 (49.5%)	87 (43%)	115 (57%)	202

*UAE, United Arab Emirates*.

The results of DTDS and PEDS were cross-tabulated for each age group to calculate test characteristics (Table 3). The prevalence of positive screening was higher for the DTDS in age groups A, B, C, D, and E, same for both tools in age group F, and the prevalence of positive cases was slightly higher for PEDS in age group G.

**Table 3:** Cross-tabulation of the Dubai Tool for Developmental Screening (DTDS) and Parent's Evaluation of Developmental Status (PEDS) results.

		**Corresponding PEDS result**		
		**Negative (%)**	**Positive (%)**	**Total (%)**
DTDS group A (9–11 months and 30 days) results	Negative (%)	185 (96.4%)	0 (0.0%)	185 (92%)
	Positive (%)	7 (3.6%)	9 (100.0%)	16 (8%)
	Total	192 (95.5%)	9 (4.5%)	201 (100%)
DTDS group B (12–14 months and 30 days) results	Negative (%)	192 (96.5%)	0 (0.0%)	192 (96.0%)
	Positive (%)	7 (3.5%)	1 (100.0%)	8 (4%)
	Total	199 (99.5%)	1 (0.5%)	200 (100%)
DTDS group C (15–17 months and 30 days) results	Negative (%)	186 (96.4%)	2 (25.0%)	188 (93.5%)
	Positive (%)	7 (3.6%)	6 (75.0%)	13 (6.5%)
	Total	193 (96%)	8 (4%)	201 (100%)
DTDS group D (18–23 months and 30 days) results	Negative (%)	216 (98.2%)	0 (0.0%)	216 (95.6%)
	Positive (%)	4 (1.8%)	6 (100.0%)	10 (4.4%)
	Total	220 (97.3%)	6 (2.7%)	226 (100%)
DTDS group E (24–29 months and 30 days) results	Negative (%)	199 (95.7%)	0 (0.0%)	199 (90.9%)
	Positive (%)	9 (4.3%)	11 (100.0%)	20 (9.1%)
	Total	208 (95%)	11 (5%)	219 (100%)
DTDS group F (30–35 months and 30 days) results	Negative (%)	180 (98.9%)	2 (22.2%)	182 (95.3%)
	Positive (%)	2 (1.1%)	7 (77.8%)	9 (4.7%)
	Total	182 (95.3%)	9 (4.7%)	191 (100%)
DTDS group G (36–48 months) results	Negative (%)	186 (98.9%)	3 (21.4%)	189 (93.6%)
	Positive (%)	2 (1.1%)	11 (78.6%)	13 (6.4%)
	Total	188 (93.1%)	14 (6.9%)	202 (100%)

*The positive and negative percentages are calculated based on the PEDS results (sensitivity and specificity), and the total percentages (prevalence) are calculated based on the total sample number in each group*.

Table 4 summarizes the sensitivity and specificity of the DTDS along with the 95% confidence intervals. The DTDS had high sensitivity and specificity in age group A (100 and 96.4%, respectively), age group B (100 and 96.5%, respectively), age group D (100 and 98.2%, respectively), and age group E (100 and 95.7%, respectively). There was moderate sensitivity and high specificity in age group C (75 and 96.4%, respectively), age group F (77.8 and 98.9%, respectively), and age group G (78.6 and 98.9%, respectively).

**Table 4:** Sensitivity, specificity, positive predictive value, negative predictive value, and kappa measure of agreement of the Dubai Tool for Developmental screening (DTDS) compared with the Parent's Evaluation of Developmental Status (PEDS) tools.

**Age group**	**Sensitivity**	**Specificity**	**Negative**	**Positive**	**Kappa**	**Standard**
	**(95% CI)**	**(95% CI)**	**predictive**	**predictive**		**Error of**
			**value**	**value**		**Cohen Kappa**
A (9–11 months and 30 days)	100% (66.4–100%)	96.4% (92.6–98.5%)	56.3%	100%	0.703	0.105
B (12–14 months and 30 days)	100% (2.5–100%)	96.5% (92.9–98.6%)	12.5%	100%	0.215	0.181
C (15–17 months and 30 days)	75% (34.5–96.8%)	96.4% (92.7–98.5%)	46.2%	98.9%	0.55	0.133
D (18–23 months and 30 days)	100% (54.1–100%)	98.2% (95.4–99.5%)	60%	100%	0.741	0.124
E (24–29 months and 30 days)	100% (71.5–100%)	95.7% (92–98%)	55%	100%	0.69	0.096
F (30–35 months and 30 days)	77.8% (40–97.2%)	98.9% (96.1–99.9%)	77.8%	99%	0.767	0.113
G (36–48 months)	78.6% (49.2–95.3%)	98.9% (96.2–99.9%)	84.6%	98.4%	0.802	0.086

*CI, confidence interval*.

The positive predictive vale was highest in age group G (84.6%) and lowest in age group B (12.5%). The negative predictive value ranged from 98.4 to 100% in all age groups. Positive predictive value and negative predictive value results are summarized in Table 4.

We calculated the kappa coefficient to assess the agreement between the DTDS and the PEDS tools to detect positive and negative cases. There was a substantial agreement between the DTDS and the PEDS tools in the age groups A (kappa = 0.703), D (kappa = 0.741), E (kappa = 0.69), F (kappa = 0.767), and G (kappa =0.802). There was moderate agreement for age group C (kappa = 0.55), and there was fair agreement for age group B (kappa = 0.215). Kappa agreement results are summarized in Table 4.

The average pairwise percent agreement within each domain revealed very high agreement between the items within each domain for all the age groups, ranging from 88.39% for the speech and language domain in age group A to 98.95% for the social and emotional domain in age group F (Table 5).

**Table 5:** Average pairwise percent agreement between the items within each domain for age groups in the Dubai Tool for Developmental screening (DTDS).

**Age group**	**Fine motor**	**Gross motor**	**Problem solving**	**Social and emotional**	**Speech and language**
A (9–11 months and 30 days)	95.69%	88.72%	95.02%	97.68%	88.39%
B (12–14 months and 30 days)	98.67%	89.33%	91%	95.67%	97%
C (15–17 months and 30 days)	94.69%	94.36%	93.37%	96.68%	88.72%
D (18–23 months and 30 days)	91.45%	96.17%	95.87%	97.35%	89.38%
E (24–29 months and 30 days)	96.06%	98.48%	94.24%	90.61%	90.30%
F (30–35 months and 30 days)	98.60%	97.21%	97.21%	98.95%	97.56%
G (36–48 months)	91.75%	94.72%	93.40%	93.40%	96.04%

## Discussion

A community-based developmental screening tool should be simple, easy to use by health care providers and parents, cost-effective and time-efficient, valid, and suit the community culture (24). The American Academy of Pediatrics (AAP) recommends comprehensive screening tools that include communication and language, fine and gross motor, personal–social, and problem-solving and behavior developmental domains. The screening tool should be culturally appropriate and available in the patient's local language (8).

The DTDS consists of 15 items with “Yes” or “No” responses, which makes it simple and less time-consuming (takes 4–8 min to finish). Parents/caregivers could answer the questionnaire while waiting for their child's well-child visit. The typical response is “Yes” to all questions, and the cut-off passing score is two “Yes” responses out of three in each domain, which makes it easy to score and interpret by health care providers and requires no special test equipment. The test can also be conducted and scored electronically by using a computer program or mobile application. The screening is comprehensive in that it examines all general developmental domains. The DTDS is among the earliest developmental screening tools developed in Arabic and the first to undergo validation in the UAE. Although initially adopted from international literature, the DTDS items were selected, filtered, and modified based on parental feedback, which reflects the characteristics of the community.

Validation studies of PEDS have used ≥ 1 predictive concern as a cut-off point to calculate sensitivity and specificity; however, in practice many physicians prefer not to proceed with additional evaluation unless there are ≥ 2 predictive concerns (39, 40). In this study, we considered ≥ 2 predictive concerns as a positive screening, but we did not ignore one predictive concern and non-predictive concern results. For these, we examined the PEDS:DM results to determine a negative/positive screening result. This makes the results more accurate and comparable to those of the DTDS. It also adheres to the “PEDS Brief Guide to Scoring and Administration” instructions, which recommend the use of a second developmental screen when one predictive concern or non-predictive concerns are present.

A screening tool is considered accurate when it has a sensitivity of 70–80% and a specificity around 80% (6, 41). In this study, the DTDS was able to detect 100% of PEDS-positive cases in age groups A, B, D, and E, while in age groups C, F, and G, the sensitivity was between 75 and 78% relative to the PEDS tools, which is still higher than the typical limit for an acceptable sensitivity, namely 70% (6, 8). The DTDS had high specificity, between 96 and 99%, in all age groups relative to the PEDS tools. Therefore, it is likely that a child who passes the DTDS is developing at a typical rate. The low positive predictive value observed in age group B, C, E and A may indicate relatively high false positive cases and hence over referral. Usually Over referral is not considered a problem in view of a screening problem, however low false negative rate is vital which means not missing any of the true positive cases, that was well achieved by the DTDS with a high predictive value over all age groups. As per Cohen's suggested kappa interpretation, we found a substantial agreement between the DTDS and PEDS tools in age groups A, D, E, F, and G, while there was a moderate agreement for age group C and a fair agreement for age group D. The high percent of agreement between the items within each domain indicates high internal reliability of the tool for all the age groups as well.

There is higher agreement, in general, in older groups than the younger groups, may be because it is easier to screen. The lowest agreement is in the age group B. That is mainly because of the difference in the number of positive cases between the two screening tests (1 (0.5) in PEDS compare with 8 (4%) in DTDS). However, this age group still maintain high sensitivity (100%) and specificity (96.5%).

This study has number of limitations. The DTDS questionnaires start from age of 9 month, though it matches the American academy of pediatrics recommendation of developmental screening stages, expansion of the tool to include younger age groups should be considered. The work team in the second stage (validation) should have been divided into two groups, both of them blinded to each other in their results so as not to lead to biased results. Although the sample size was sufficient to compare screening results of the DTDS to those of the PEDS tools, the absolute number of children with positive screening was relatively low, particularly in age groups B and D. Hence, conducting a study with more participants would be preferable to verify the findings. In this study, the DTDS was validated against a valid screening tool (PEDS). Using a diagnostic tool would identify children with developmental delay more accurately. Repeating the study against a validated diagnostic developmental tool should be considered in future research.

## Conclusion

The study findings have demonstrated that the DTDS is a valid screening tool for the early identification of developmental delays and disabilities in infants and children aged 9–48 months. It is simple, quick to administer and score, and culturally relevant to the UAE population.

## Data availability statement

The raw data supporting the conclusions of this article will be made available by the authors, without undue reservation. Restrictions apply to the availability of the DTDS questionnaires, which require permission from DHA.

## Ethics statement

The study protocol was reviewed and approved by the Dubai Scientific Research Ethics Committee (DSREC), Dubai Health Authority, (Approval Number DSREC-10/2018_06). The patients/participants provided their written informed consent to participate in this study.

## Author contributions

FA and HM prepared design and methodology of the study. FA, HM, and IA wrote the initial draft of the manuscript. All authors contribute writing the final manuscript. All authors contributed to the article and approved the submitted version.

## Conflict of interest

The authors declare that the research was conducted in the absence of any commercial or financial relationships that could be construed as a potential conflict of interest.

## Publisher's note

All claims expressed in this article are solely those of the authors and do not necessarily represent those of their affiliated organizations, or those of the publisher, the editors and the reviewers. Any product that may be evaluated in this article, or claim that may be made by its manufacturer, is not guaranteed or endorsed by the publisher.

## Abbreviations

DTDS: Dubai Tool for Developmental Screening
PEDS: Parent's Evaluation of Developmental Status
PEDS:DM: Parent's Evaluation of Developmental Status: Developmental Milestones
DHA: Dubai Health Authority
UAE: United Arab Emirates.

## References

[1] Rosenberg SA Zhang D Robinson CC. Prevalence of developmental delays and participation in early intervention services for young children. Pediatrics. (2008) 121:e1503–9. 10.1542/peds.2007-1680 18504295

[2] Boyle CA Decouflé P Yeargin-Allsopp M. Prevalence and health impact of developmental disabilities in US children. Pediatrics. (1994) 93:399–403. 10.1542/peds.93.3.399 7509480

[3] Roberts MY Kaiser AP. Early intervention for toddlers with language delays: a randomized controlled trial. Pediatrics. (2015) 135:686–93. 10.1542/peds.2014-2134 25733749 PMC4379460

[4] Campbell FA Pungello EP Miller-Johnson S Burchinal M Ramey CT. The development of cognitive and academic abilities: growth curves from an early childhood educational experiment. Dev Psychol. (2001) 37:231–42. 10.1037/0012-1649.37.2.231 11269391

[5] Ramey CT Ramey SL. Early intervention and early experience. Am Psychol. (1998) 53:109–20. 10.1037/0003-066X.53.2.109 9491742

[6] Glascoe FP. Screening for developmental and behavioral problems. Ment Retard Dev Disabil Res Rev. (2005) 11:173–9. 10.1002/mrdd.20068 16161092

[7] Aylward GP. Developmental screening and assessment: what are we thinking? J Dev Behav Pediatr. (2009) 30:169–73. 10.1097/DBP.0b013e31819f1c3e 19363370

[8] Developmental Behavioral Pediatrics B. Medical home initiatives for children with special needs project advisory committee. Identifying infants and young children with developmental disorders in the medical home: an algorithm for developmental surveillance and screening. Pediatrics. (2006) 118:405–20. 10.1542/peds.2006-1231 16818591

[9] Hamilton S. Screening for developmental delay: reliable, easy-to-use tools. J Fam Pract. (2006) 55:415–22. 16670037

[10] Rydz D Srour M Oskoui M Marget N Shiller M Birnbaum R. Screening for developmental delay in the setting of a community pediatric clinic: a prospective assessment of parent-report questionnaires. Pediatrics. (2006) 118:e1178–86. 10.1542/peds.2006-0466 17015506

[11] Oberklaid F Efron D. Developmental delay–identification and management. Aust Fam Physician. (2005) 34:739–42. 16184205

[12] Cunningham PJ. Beyond parity: primary care physicians' perspectives on access to mental health care. Health Aff. (2009) 28:w490–501. 10.1377/hlthaff.28.3.w490 19366722

[13] Horwitz SM Kelleher KJ Stein REK Storfer-Isser A Youngstrom EA Park ER. Barriers to the identification and management of psychosocial issues in children and maternal depression. Pediatrics. (2007) 119:e208–18. 10.1542/peds.2005-1997 17200245

[14] Weitzman C Wegner L Section Section on Developmental and Behavioral Pediatrics Committee Committee on Psychosocial Aspects of Child and Family Health Council Council on Early Childhood Society for Developmental and Behavioral Pediatrics . Promoting optimal development: screening for behavioral and emotional problems. Pediatrics. (2015) 135:384–95. 10.1542/peds.2015-0904 25624375

[15] Ertem IO Dogan DG Gok CG Kizilates SU Caliskan A Atay G. A guide for monitoring child development in low- and middle-income countries. Pediatrics. (2008) 121:e581–9. 10.1542/peds.2007-1771 18310178

[16] Gladstone MJ Lancaster GA Jones AP Maleta K Mtitimila E Ashorn P. Can Western developmental screening tools be modified for use in a rural Malawian setting? Arch Dis Child. (2008) 93:23–9. 10.1136/adc.2006.095471 17379661

[17] Early Detection Tools for Children With Developmental Delays And Disabilities. Unicef.org. (2022). Available online at: https://www.unicef.org/mena/reports/early-detection-tools-children-developmental-delays-and-disabilities (accessed June 25, 2022).

[18] Page FP. Parents evaluation of developmental status (PEDS). Indian Pediatr. (2003) 40:439–42. 12768055

[19] Glascoe FP. Using parents' concerns to detect and address developmental and behavioral problems. J Soc Pediatr Nurses. (1999) 4:24–35. 10.1111/j.1744-6155.1999.tb00077.x 10334009

[20] Glascoe FP. Collaborating With Parents: Using Parents' Evaluation of Developmental Status to Detect and Address Developmental and Behavioral Problems. 2nd ed. Nashville, TN: Ellsworth & Vandermeer Press (2013).

[21] Squires J Bricker DD Twombly E. Ages and Stages Questionnaires. Baltimore: Paul H. Brookes (2009).

[22] Singh A Yeh CJ Boone Blanchard S. Ages and stages questionnaire: a global screening scale. Bol Med Hosp Infant Mex. (2017) 74:5–12. 10.1016/j.bmhimx.2016.07.008 29364814

[23] Fischer VJ Morris J Martines J. Developmental screening tools: feasibility of use at primary healthcare level in low- and middle-income settings. J Health Popul Nutr. (2014) 32:314–26. 25076668 PMC4216967

[24] Chopra G Verma IC Seetharaman P. Development and assessment of a screening test for detecting childhood disabilities. Indian J Pediatr. (1999) 66:331–5. 10.1007/BF02845517 10798079

[25] Lung FW Shu BC Chiang TL Lin SJ. Efficient developmental screening instrument for 6- and 18-month-old children in the Taiwan Birth Cohort Pilot Study. Acta Paediatr. (2008) 97:1093–8. 10.1111/j.1651-2227.2008.00832.x 18462464

[26] Gladstone M Lancaster GA Umar E Nyirenda M Kayira E van den Broek NR. The Malawi Developmental Assessment Tool (MDAT): the creation, validation, and reliability of a tool to assess child development in rural African settings. PLoS Med. (2010) 7:e1000273. 10.1371/journal.pmed.1000273 20520849 PMC2876049

[27] El Shafie AM Omar ZAL Bashir MM Mahmoud SF Basma EM Hussein AE. Development and validation of Egyptian developmental screening chart for children from birth up to 30 months. PeerJ. (2020). 8:e10301. 10.7717/peerj.10301 33240634 PMC7666562

[28] Balasundaram P Avulakunta I. Bayley Scales Of Infant Toddler Development. Ncbi.nlm.nih.gov (2022). Available online at: https://www.ncbi.nlm.nih.gov/books/NBK567715/ (accessed 25 June 2022).

[29] Phatak AT Khurana B. Baroda development screening test for infants. Indian Pediatr. (1991) 28:31–7. 1711514

[30] Frankenburg WK Dodds J Archer P Shapiro H Bresnick B. The Denver II: a major revision and restandardization of the denver developmental screening test. Pediatrics. (1992) 89:91–7. 10.1542/peds.89.1.91 1370185

[31] Nair MK Nair GS George B Suma N Neethu C Leena ML. Development and validation of Trivandrum Development Screening Chart for children aged 0-6 years [TDSC (0-6)]. Indian J Pediatr. (2013) 80 (Suppl. 2):S248–55. 10.1007/s12098-013-1144-2 24014206

[32] Nair MK Russell PS Rekha RS Lakshmi MA Latha S Rajee K. Validation of developmental assessment tool for Anganwadis (DATA). Indian Pediatr. (2009). 46:s27–36. 19279366

[33] Abubakar A Holding P van Baar A Newton CR van de Vijver FJ. Monitoring psychomotor development in a resource-limited setting: an evaluation of the Kilifi Developmental Inventory. Ann Trop Paediatr. (2008) 28:217–26. 10.1179/146532808X335679 18727851 PMC3908377

[34] Glascoe FP Robertshaw NS. PEDS: Developmental Milestones. A Tool for Surveillance and Screening. 2nd ed. Nashville, TN: Ellsworth & Vandermeer Press (2010). 10.1177/0009922807309419 18057141

[35] Sample Size Software Power Analysis Software PASS. Ncss.com (2018). Available online at: https://www.ncss.com/software/pass/ (accessed February 20, 2019).

[36] MedCalc, Software Ltd,. Free Statistical Calculators. Available online at: https://www.medcalc.org/calc/diagnostic_test.php (accessed November 26, 2019).

[37] Landis JR Koch GG. An application of hierarchical kappa-type statistics in the assessment of majority agreement among multiple observers. Biometrics. (1977) 33:363–74. 10.2307/2529786 884196

[38] Lorenzo-Seva U Ferrando PJ. TETRA-COM: a comprehensive SPSS program for estimating the tetrachoric correlation. Behav Res Methods. (2012) 44:1191–6. 10.3758/s13428-012-0200-6 22477439

[39] Sices L Stancin T Kirchner L Bauchner H PEDS. and ASQ developmental screening tests may not identify the same children. Pediatrics. (2009) 124:e640–647. 10.1542/peds.2008-2628 19736268 PMC2764374

[40] Glascoe FP editor. Collaborating With Parents: Using Parents' Evaluations of Developmental Status to Detect and Address Developmental and Behavioral Problems. Nashville, TN: Ellsworth & Vandermeer Press (2002).

[41] Rydz D Shevell MI Majnemer A Oskoui M. Developmental screening. J Child Neurol. (2005) 20:4–21. 10.1177/08830738050200010201 15791916

