# Supplementary material for: Designing and validating the Dubai Tool for Developmental Screening (DTDS)
Source: Front Pediatr. 2022 Aug 22;10:924017. doi: 10.3389/fped.2022.924017 (PMC9441853; doi:10.3389/fped.2022.924017)
Supplement: Supplementary file 1 [file Data_Sheet_1.PDF]

DUBAI TOOL FOR DEVELOPMENTAL SCREENING

Form C (15 months -17 months 30 days)

Child's Name:				Nationality:	
Date of Birth:		Child's Age:		MRN:	

Information of the person filling the questionnaire

Name:			
Relationship with child:		Today's Date:	

Corrected age (to be filled by staff):	

Please answer these questions about your child. Please circle **YES** or **NO** for every question.

GROSS MOTOR

• Does your child walk without falling much?	**YES**	**NO**
• Does your child climb on furniture?	**YES**	**NO**
• Does your child bend over to pick up an object from the floor and stand up? (may be with one hand supported)	**YES**	**NO**

FINE MOTOR

• Does your child pick up a small object like a raisin, using only the ends of his thumb and index finger?	**YES**	**NO**
• Does your child take her/his socks or shoes off if you undo the laces?	**YES**	**NO**
• Does your child try to use a spoon?	**YES**	**NO**

SPEECH AND LANGUAGE

• Does your child follow one step command without gesture? E.g., come here	**YES**	**NO**
• Does your child nod or say YES?	**YES**	**NO**
• Does your child say three words with meaning?	**YES**	**NO**

SOCIAL AND EMOTIONAL

• Does your child hug you back if you hug her/him?	**YES**	**NO**
• Does your child bring toys to you when need help to operate?	**YES**	**NO**

• Does your child show empathy (looks sad when someone else cries)?	**YES**	**NO**

PROBLEM SOLVING AND SELF-HELP

• Does your child try with toys to make them work?	**YES**	**NO**
• Does your child use a spoon to eat (may spill some)?	**YES**	**NO**
• Does your child try to brush own hair?	**YES**	**NO**

Thank you
