# Supplementary material for: Designing and validating the Dubai Tool for Developmental Screening (DTDS)
Source: Front Pediatr. 2022 Aug 22;10:924017. doi: 10.3389/fped.2022.924017 (PMC9441853; doi:10.3389/fped.2022.924017)
Supplement: Supplementary file 2 [file Data_Sheet_2.PDF]

أداة دبي لفحص التطور النمائي (15-17 شهر) Form C

**اسم الطفل**			
**تاريخ الولادة**		**العمر**	
**الجنسية**		**رقم البطاقة الصحية**	

معلومات الشخص الذي يملأ الاستمارة:

**الاسم**			
**صلة القرابة**		**رقم الاتصال**	

**تصحيح العمر بالنسبة للخداجة (تملأ من قبل الطاقم الطبي):**	

من فضلك أجب عن هذه الأسئلة الخاصة بطفلك بوضع دائرة حول **نعم** أو **لا** لكل سؤال

مهارات الحركات الكبيرة (GROSS MOTOR)

• هل يمشي طفلك دون أن يسقط كثيراً؟	**نعم**	**لا**
• هل يتسلق طفلك الأثاث؟	**نعم**	**لا**
• هل ينحني طفلك ليلتقط شيء من الأرض ثم يقف (قد يستند بإحدى يديه)؟	**نعم**	**لا**

مهارات الحركات الدقيقة (FINE MOTOR)

• هل يلتقط طفلك الأشياء الصغيرة مثل حبة الزبيب بإصبعيه السبابة والابهام؟	**نعم**	**لا**
• هل يخلع طفلك شرابه أو حذاءه (إذا فككت له الرباط)؟	**نعم**	**لا**
• هل يحاول طفلك استخدام الملعقة؟	**نعم**	**لا**

مهارات الكلام (SPEECH AND LANGUAGE)

• هل يتبع طفلك أمرا من خطوة واحدة دون ان تؤشري له؟ مثال (تعال الى هنا)	**نعم**	**لا**
• هل يقول طفلك كلمة "نعم" أو يومئ برأسه للموافقة؟	**نعم**	**لا**
• هل يقول طفلك ثلاث كلمات (او أكثر) ذات معنى؟	**نعم**	**لا**

المهارات الاجتماعية/ العاطفية (SOCIAL AND EMOTIONAL)

• هل يقوم طفلك بمعانقتك إذا عانقته؟	**نعم**	**لا**

• هل يحضر طفلك اللعب إليك لتساعده في تشغيلها؟	**نعم**	**لا**
• هل يظهر طفلك التعاطف مثال (يبدو حزينا عندما يبكي شخص آخر)؟	**نعم**	**لا**

مهارات حل المشكلات/ الاعتماد على النفس (PROBLEM SOLVING AND SELF-HELP)

• هل يحاول طفلك جعل لعبة تعمل (يحاول تشغيلها)؟	**نعم**	**لا**
• هل يستخدم طفلك الملعقة للأكل (قد يسقط بعض الطعام منها)؟	**نعم**	**لا**
• هل يحاول طفلك تمشيط شعره بنفسه؟	**نعم**	**لا**

شكرا لكم
